# Pectins that Structurally Differ in the Distribution of Methyl‐Esters Attenuate *Citrobacter rodentium*‐Induced Colitis

**DOI:** 10.1002/mnfr.202100346

**Published:** 2021-08-16

**Authors:** Martin Beukema, Renate Akkerman, Éva Jermendi, Taco Koster, Anne Laskewitz, Chunli Kong, Henk A. Schols, Marijke M. Faas, Paul de Vos

**Affiliations:** ^1^ Immunoendocrinology Division of Medical Biology Department of Pathology and Medical Biology University Medical Center Groningen Groningen The Netherlands; ^2^ Laboratory of Food Chemistry Wageningen University and Research Wageningen The Netherlands

**Keywords:** *Citrobacter rodentium*, colitis, degree of blockiness, degree of methyl‐esterification, microbiota, pectin

## Abstract

**Introduction:**

Pectins have anti‐inflammatory properties on intestinal immunity through direct interactions on Toll‐like receptors (TLRs) in the small intestine or via stimulating microbiota‐dependent effects in the large intestine. Both the degree of methyl‐esterification (DM) and the distribution of methyl‐esters (degree of blockiness; DB) of pectins contribute to this influence on immunity, but whether and how the DB impacts immunity through microbiota‐dependent effects in the large intestine is unknown. Therefore, this study tests pectins that structurally differ in DB in a mouse model with *Citrobacter rodentium* induced colitis and studies the impact on the intestinal microbiota composition and associated attenuation of inflammation.

**Methods and Results:**

Both low and high DB pectins induce a more rich and diverse microbiota composition. These pectins also lower the bacterial load of *C. rodentium* in cecal digesta. Through these effects, both low and high DB pectins attenuate *C. rodentium* induced colitis resulting in reduced intestinal damage, reduced numbers of Th1‐cells, which are increased in case of *C. rodentium* induced colitis, and reduced levels of GATA3^+^ Tregs, which are related to tissue inflammation.

**Conclusion:**

Pectins prevent *C. rodentium* induced colonic inflammation by lowering the *C. rodentium* load in the caecum independently of the DB.

## Graphical abstract

Pectins with a low or a high degree of blockiness (DB) both increase microbial diversity and low *C. rodentium* load in cecal digesta. The pectins protected independent of the DB from *C. rodentium*‐induced barrier disruption, intestinal damage and inflammation.

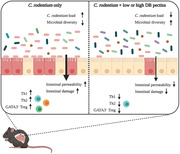

## 1 Introduction

Dietary fibers influence health through immune‐modulating effects,^[^ ^1^, ^2^, ^3^, ^4^, ^5^, ^6^ ^]^ but the exact mechanism explaining the impact of dietary fibers on immunity is not fully understood. It is believed that dietary fibers impact intestinal immunity through direct interaction with immune cells, or indirectly through changing the composition or function of gut microbiota.^[^ ^7^ ^]^ Dietary fibers can stimulate intestinal immunity through stimulating the growth of commensals and expose the intestinal immune system to microbial‐derived molecules with immune‐modulating effects, such as lipoteichoic acid, exopolysaccharides, or short‐chain fatty acids (SCFA).^[^ ^8^, ^9^ ^]^ Additionally, dietary fibers can also preserve intestinal integrity by preventing pathogenic infection and damage by enhancing the growth of commensal communities that compete with pathogens, by having anti‐microbial effects or through decoy effects.^[^ ^10^, ^11^ ^]^ Microbiota‐dependent effects occur mainly in the large intestine since this intestinal region contains a high abundance of microbiota and a thick mucus layer that prevents direct interactions with intestinal immunity. However, direct effects of dietary fibers occur more at small‐intestinal sites, as it contains a loose mucus layer and a low abundance of the intestinal microbiota.^[^ ^7^ ^]^

Pectin is a dietary fiber with a known impact on intestinal immunity, but this impact of pectin is strongly dependent on its chemical characteristics.^[^ ^12^, ^13^, ^14^ ^]^ Commercial pectins mainly consist of homogalacturonan regions (>70%) that is composed of α‐1‐4 galacturonic acids (GalA) molecules, which form the galacturonic acid backbone. Commercial pectins may also contain other regions in lower abundance, such as Rhamnogalacturonan I and Rhamnogalacturonan II.^[^ ^15^ ^]^ The GalA molecules in the homogalacturonan regions can be methyl‐esterified as expressed by the degree of methyl‐esterification (DM).^[^ ^16^ ^]^ Methyl‐esters present within the GalA backbone can also be differently distributed which is indicated by the degree of blockiness (DB). Pectins with a high DB have a more blockwise distribution of non‐esterified GalA residues, whereas pectins with a low DB have a more random distribution of non‐esterified GalA residues.^[^ ^17^ ^]^ The DM and DB of pectins were essential in the direct effects on TLR2‐1 as the anti‐inflammatory properties of TLR2‐1 were only observed with low DM pectins or intermediate DM (∼DM46) pectins with a higher DB.^[^ ^18^ ^]^ The DM and DB were essential in anti‐inflammatory effects of pectins on TLR2‐mediated inflammatory responses in vitro and in mice.^[^ ^14^, ^18^ ^]^ Additionally, the DM also played an important role in the stimulation of intestinal microbiota communities in several in vitro and in vivo models.^[^ ^19^, ^20^, ^21^ ^]^ How the DB of pectins impacts the microbiota‐dependent modulation of intestinal immunity is unknown.

A way to investigate how stimulation of microbiota composition impacts intestinal immunity is by applying an enteric infection of *Citrobacter rodentium* in mice.^[^ ^22^ ^]^ *C. rodentium* is the murine equivalent of the human Enteropathogenic *Escherichia coli* (EPEC) and Enterohemorrhagic *Escherichia coli* (EHEC), because they share the same virulence mechanism.^[^ ^22^ ^]^ These three enteric pathogens can attach to the intestinal epithelium, which leads to the formation of attaching and effacing (A/E) lesions^[^ ^22^ ^]^ and the development of colitis that is characterized by crypt elongation, goblet cell depletion, cytokine responses from innate immune cells, and the induction of T cell responses.^[^ ^22^ ^]^ This colitis is accompanied by large alterations of the intestinal microbiota composition in mice that is characterized by an overgrowth of the *C. rodentium*.^[^ ^23^ ^]^ Stimulation of the intestinal microbiota with prebiotics or probiotics have been shown to reduce this *C. rodentium* induced microbiota alterations and reduces the development of colitis.^[^ ^10^, ^24^, ^25^ ^]^

Since pectins are known to stimulate alterations in intestinal microbiota composition,^[^ ^20^, ^21^ ^]^ we investigated whether pectins that structurally differ in the DB prevent the development of *C. rodentium* induced colitis through stimulation of the intestinal microbiota composition. Two high DM pectins (∼DM60) that differed in DB were tested. Mice were fed for 7 days with pectins prior to *C. rodentium* inoculation. On day 7 of *C. rodentium* infection, mice were sacrificed and intestinal histology, barrier function, and inflammation were investigated. The impact of pectins on the intestinal microbiota communities and SCFA production was also determined.

## 2 Experimental Section

### 2.1 Pectins

Two commercially extracted pectins from orange peel were used in the current study (Andre Pectin Co. Ltd., Yantai, China). Molecular weight, sugar composition, degree of methyl‐esterification (DM), and degree of blockiness (DB) of these pectins were determined as previously described.^[^ ^18^ ^]^ Two high DM pectins were used to study the impact of DB as the difference in distribution of non‐esterified GalA residues is larger at this DM compared to low DM. Low DM pectins generally possess a high number blockwise distributed non‐esterified GalA residues (high DB) due to the low level of methyl‐esterified GalA.^[^ ^17^ ^]^ Therefore, pectins with a low DM that have different DB do structurally not differ much from each other.

### 2.2 Mice

C57BL6 female mice (10 weeks old) were obtained from Janvier Laboratories (Le Genest‐Saint‐Isle, France). The experimental use of animals was approved by Animal Ethical Committee of the University of Groningen (CCD application number AVD1050020171487). All mice were acclimatized for 1.5 weeks prior to the start of the experiment. Mice were co‐housed in individual ventilated cages and cohoused with a total number of five mice from similar experimental groups. Control groups were fed ad libitum with RHB‐B diet (AB Diets, Woerden, The Netherlands). Pectin treated mice were fed ad libitum with RHB‐B chow, containing 5% (w/w, human equivalent dose of 8.3 g kg^−1^) of one of the pectins. Dosage was chosen based on previous studies.^[^ ^26^, ^27^ ^]^

### 2.3 *C. rodentium* Infection

Mice were fed for 14 days with normal chow or chow containing pectins. After 7 days, 100 µL of *Citrobacter rodentium* DBS‐100 (American Type Tissue Culture, Manassas, USA) in LB agar (Sigma Aldrich, Zwijndrecht, The Netherlands) was orally administered in a dose of 10^9^ CFU mL^−1^ to the mice. Control mice received oral administration of LB agar only. Bodyweight and disease activity score (ranging from 0 to 11) were monitored daily after *C. rodentium* administration as previously described.^[^ ^28^ ^]^ At day 14, FITC dextran (4kDa, TdB Consultancy AB, Uppsala, Sweden) was orally administered [600 mg kg^−1^ in PBS (Lonza, Basel, Switzerland)] to measure large intestinal barrier functioning.^[^ ^29^ ^]^ After 4 h, mice were anesthetized with isoflurane/O_2_ and blood was collected in 50 µL EDTA after heart punction. Mice were terminated by cervical dislocation. Blood was centrifuged at 12 000 x *g* and plasma was stored at ‐80°C until further analysis. Colon tissues were collected for histological analysis and mesenteric lymph nodes (MLN) were collected to measure T cell frequencies. Cecal tissues were collected to measure tissue cytokines. Cecal digesta was snap frozen in liquid nitrogen and stored at ‐80 °C until microbiota and SCFA analysis.

### 2.4 Intestinal Barrier Function

FITC‐dextran was measured from plasma to determine the paracellular transport of FITC‐dextran over the intestinal barrier. Plasma was diluted in an equal volume of PBS. Standards (50‐0.156 µg mL^−1^) were also diluted in PBS. Samples and standards were transferred to a black 96‐wells plate (Costar, Corning incorporate, USA) in a volume of 100 µL. PBS only was used as blank. FITC‐dextran analysis was performed with a microplate spectrophotometer (Clariostar, De Meern, The Netherlands) at an excitation wavelength of 485 nm and an emission wavelength of 528 nm.

### 2.5 Histology

Colon samples were incubated for 24 h in 4% paraformaldehyde in PBS and embedded in paraffin. Paraffin sections were cut at 4 µM and H&E staining was performed on the slices. The stained slides were scanned at a magnification of 40x using a Hamamatsu slide scanner (Hamamatsu Photonics, Hamamatsu, Japan).

### 2.6 Cecal Cytokine Measurements

Cecal cytokines were determined as described before.^[^ ^27^ ^]^ Briefly, after collection from mice, cecal tissues were washed in PBS. Next, tissues were transferred to 2 mL ice‐cold PBS containing 1x protease inhibitor cocktail (Sigma Aldrich) and homogenized using cell homogenizer for 2 min (Tamson, Zoetermeer, The Netherlands). After this, the cell suspension was centrifuged at 12 000 x *g* and the supernatant was collected for protein determination and cytokine measurements. Protein determination was quantified using BCA protein assay kit (Thermo Scientific, Etten‐Leur, The Netherlands). TNF‐α, IL‐6, and IL‐10 levels from cecal cell lysates were determined with ELISA (R&D systems, Minneapolis, USA) according to manufacturer's instructions. Cytokine levels were corrected for the protein levels in the cell lysates.

### 2.7 T Cell Staining and Flow Cytometry

Cells from MLN were isolated as previously described.^[^ ^30^ ^]^ To stain T cells, 1 × 10^6^ MLN cells were transferred to a 96 wells plate and centrifuged for 5 min. The cells were washed once with PBS and incubated for 15 min with ZombieNIR (**Table** **1**). After this step, the cells were washed with FACS buffer (PBS + 2% dFCS) and incubated for 10 min with extracellular blocking buffer containing 20% (v/v) rat serum (Jackson, Newmarket, UK), 78% (v/v) FACS buffer and 2% (v/v) FC block (eBioscience, Vienna, Austria). Next, cells were incubated with 25 µL extracellular antibody mix (Table 1) for 30 min. Then the cells were washed with FACS buffer and incubated for 30 min with FACS lysing buffer (BD Biosciences, Breda, the Netherlands) containing 2% PFA to fixate the cells. Next, the cells were washed twice with permeabilization buffer consisting of demi water + 5% (v/v) PERM (Invivogen, Toulouse, France). Then the cells were intracellularly blocked for 10 min with intracellular blocking buffer (20% (v/v)) normal rat serum in permeabilization buffer. After this step, cells were incubated for 30 min with 50 µL intracellular antibody mix (Table 1). This was followed by two washing steps with permeabilization buffer. Finally, the cells were resuspended in 100 µL FACS buffer and stored at 4°C until analysis within 16 h. In all centrifugation steps, cells were centrifuged at 600 x *g* for 3 min at 4°C. Incubation steps were performed on ice. FMO controls were used to set the gates (Supplementary Figure S1, Supporting Information).

**Table 1:** Extracellular and intracellular antibody mix T cell staining

Marker	Fluorchrome	Antibody mix	Dilution	Company (catalogue number)
CD3	BV605	Extracellular	25x	Biolegend (100 237)
CD4	CD4‐PE‐Cy7	Extracellular	100x	Biolegend (100 422)
CD8	CD8‐ PerCP‐Cy5.5	Extracellular	50x	Biolegend (100 734)
Tbet	BV421	Intracellular	10x	Biolegend (644 816)
GATA3	AF647	Intracellular	100x	BD (560 068)
Rorγt	PE	Intracellular	100x	eBioscience (12‐6981‐80)
Foxp3	FITC	Intracellular	50x	eBioscience (11‐5773‐82)
dead/live	Zombie NIR	‐	1000x	Biolegend (423 105)

The FACSverse flow cytometer system was used to measure the samples (BD Biosciences Franklin Lakes, USA), using the FACSsuite software. Analysis was performed by FCS Express software version 6 (De Novo Software, Pasadena, USA).

### 2.8 DNA Extraction Cecal Digesta for Microbiota Analysis

Cecal digesta was collected and immediately snap‐frozen in screw caps in liquid nitrogen and stored −80°C until DNA isolation. For DNA isolation approximately 0.25 g of digesta was collected and DNA was isolated using QIAmp Powerfecal DNA kit (QIAGEN, Hilden, Germany). DNA concentration was measured with NanoDrop ND‐1000 Spectrophotometer (Thermo Fisher Scientific, Waltham, MA, USA). Digesta mass was weighted before each isolation step.

### 2.9 16S rRNA Gene Sequencing, Quality Control, and Taxonomy Assignment

Samples were sent to NOVOGENE (Cambridge, UK) for sequencing, quality control, and taxonomy assignment. In short, DNA was used for the amplification of the V3‐V4 region of bacterial 16S rRNA using 341F primer (5’‐CCTAYGGGRBGCASCAG‐3’) and 806R primer (5’‐GGACTACNNGGGTATCTAAT‐3’) containing a six‐nucleotide barcode (Novogenes, Cambridge, UK). The PCR product was then selected for proper size and purified for library preparation. The same amount of PCR product from each sample was pooled, end polished, A‐tailed, and ligated with adapters. After purification, the library was analyzed for size distribution, quantified using real‐time PCR, and sequenced on NovaSeq 6000 SP flowcell with PE250.

Sequences analysis was performed by Uparse software (Uparse v7.0.1001 http://drive5.com/uparse/) using all the effective tags. Sequences with ≥97% similarity were assigned to the same operational taxonomic units (OTUs). Representative sequence for each OTU was screened for further annotation. For each representative sequence, Mothur software was performed against the SSUrRNA database of SILVA Database (http://www.arb‐silva.de/) for species annotation at each taxonomic rank (Threshold: 0.8∼1) (kingdom, phylum, class, order, family, genus, species). Alpha diversity, including shannon index and Chao1 index, or Beta diversity on both weighted and unweighted unifrac were calculated with QIIME (Version 1.7.0) and displayed with Graphpad software (Version 8.4.1).

### 2.10 Real‐time PCR on Cecal Digesta to Determine *C. rodentium* Load

To measure *C. rodentium* load in the caecal digesta, real‐time PCR was performed on DNA that was isolated from the cecal digesta. DNA was also isolated from *C. rodentium* cultures using QIAmp Powerfecal DNA kit (QIAGEN) to make a standard of 0 – 2.1×10^10^ CFU. Forward primer 5’‐ATGCCGCAGATGAGACAGTTG‐3’ and reverse primer 5’‐ GTCAGCAGCCTTTTCAGCTA‐3’ were previously designed and specific for EspB gene of *C. rodentium*.^[^ ^31^ ^]^ Next, isolated DNA from standards and 12.5 ng cecal DNA was mixed with 10 mM primers and with SYBRgreen mastermix (Roche, Basel, Switzerland). The reaction was started with an enzyme activation step of 10 min at 95°C. Then, the amplification reaction was performed for 40 cycles, starting with denaturation for 15 s at 95°C, followed by annealing for 60 s at 60°C, and extension for 30 s at 72°C in a ViiA Real‐time PCR System (Applied Biosystems, Foster City, CA, USA). *C. rodentium* load in CFU was calculated from CT values of the standard curve and corrected to CFU/gram by using digesta mass that was measured for DNA isolation.

### 2.11 SCFA Profiling

For SCFA analysis, 20–150 mg caecum content was diluted in 150 µL ultrapure water, mixed, and centrifuged at 20 000 x *g* for 10 min. Next, 100 µL supernatant was used for SCFA analysis using a Dionex Ultimate 3000 HPLC (Thermo Scientific, Dionex, Sunnyvale, CA, USA). Then, 10 µL sample was injected to an ion‐exclusion Aminex HPX‐87H column (7.8×300 mm) combined with a guard column (Bio‐Rad, Hercules CA, USA). The elution was monitored by refractive index detection (Shodex RI 101; Showa Denko K.K., Tokyo, Japan). Elution was done with a flow rate of 0.6 mL min^−1^ using 5.0 mM H_2_SO_4_ at 65°C.^[^ ^32^ ^]^ Standard solutions of lactic acid, succinic acid, acetic acid, propionic acid, butyric acid, isovaleric acid, and isobutyric acid were prepared in concentrations of 0.03125–2 mg mL^−1^. Data were processed using Chromeleon 7.2 (Thermo Scientific, Dionex, Sunnyvale, CA, USA). SCFA concentrations were expressed µmol mg^−1^ dry matter to correct for the potential impact of digest consistency. Dry matter content was determined by drying the samples in an oven overnight at 60°C.

### 2.12 Statistics

Past3 software was used for Permanova, and Simper similarity test.^[^ ^33^ ^]^ Graphpad software (Version 8.4.1) (La Jolla, CA, USA) was used to measure statistical differences for crypt depth, intestinal barrier function, cecal cytokine production, T cell frequencies, and individual bacterial families and species. Normal distribution was confirmed using the Kolmogorov‐Smirnov test. Statistical differences between control, *C. rodentium*, *C. rodentium* + DM59 (low DB) pectin, *C. rodentium* + DM64 (high DB) pectin were tested with *t*‐test (comparison two groups) and one‐way ANOVA (comparison multiple groups) for parametrically distributed data and with Mann‐Whitney (comparison two groups) and Kruskal‐Wallis (comparison multiple groups) for non‐parametrically distributed data. Post‐testing was performed with Dunnet to test statistical differences between *C. rodentium* and pectin treated mice (*p* < 0.05 was considered as statistically significant; * *p* < 0.05, ** *p* < 0.01, *** *p* < 0.001, **** *p* < 0.0001). *p* between 0.05 and 0.10 were considered as a trend. *p*‐values of microbiota data were corrected for multiple testing by the false discovery rate (FDR) of Benjamini–Hochberg (FDR < 0.05).

## 3 Results

### 3.1 Structural Characteristics of Pectins

Pectins were characterized for the degree of methyl‐esterification (DM), degree of blockiness (DB), molecular weight, and sugar composition (**Table** **2**). The homogalacturonan pectins were rather similar in sugar composition and molecular weight. The pectins also had a similar DM range (59% and 64%), but they did differ in DB. The DM59 pectin had a DB of 17%, whereas the DM64 pectin had a higher DB of 37%. These findings demonstrate that both pectins possess around 40% non‐esterified GalA residues of which 37% are distributed in blocks in the DM64 pectin and 17% of the non‐esterified GalA residues are distributed in blocks in the DM59 pectin.

**Table 2:** Structural characteristics of pectins

Pectin	origin	DB [%]	Mw	Sugar composition [mol%]					Carbohydrate content [%]
				Rha	Ara	Gal	Glc	UA	
DM59	orange	17	86 000	1	3	8	1	88	87
DM64	orange	37	92 000	1	7	8	2	82	81

Degree of methyl‐esterification (DM), Degree of blockiness (DB), Weight‐average molecular weight (Mw), and sugar composition of the pectins. Rha, rhamnose; Ara, arabinose; Gal, Galactose; Glc, Glucose; UA, Uronic Acid.

### 3.2 Both Low DB and High DB Pectins Exert a Protective Effect Against *C. rodentium*‐Induced Colonic Damage

**Figure 1:**
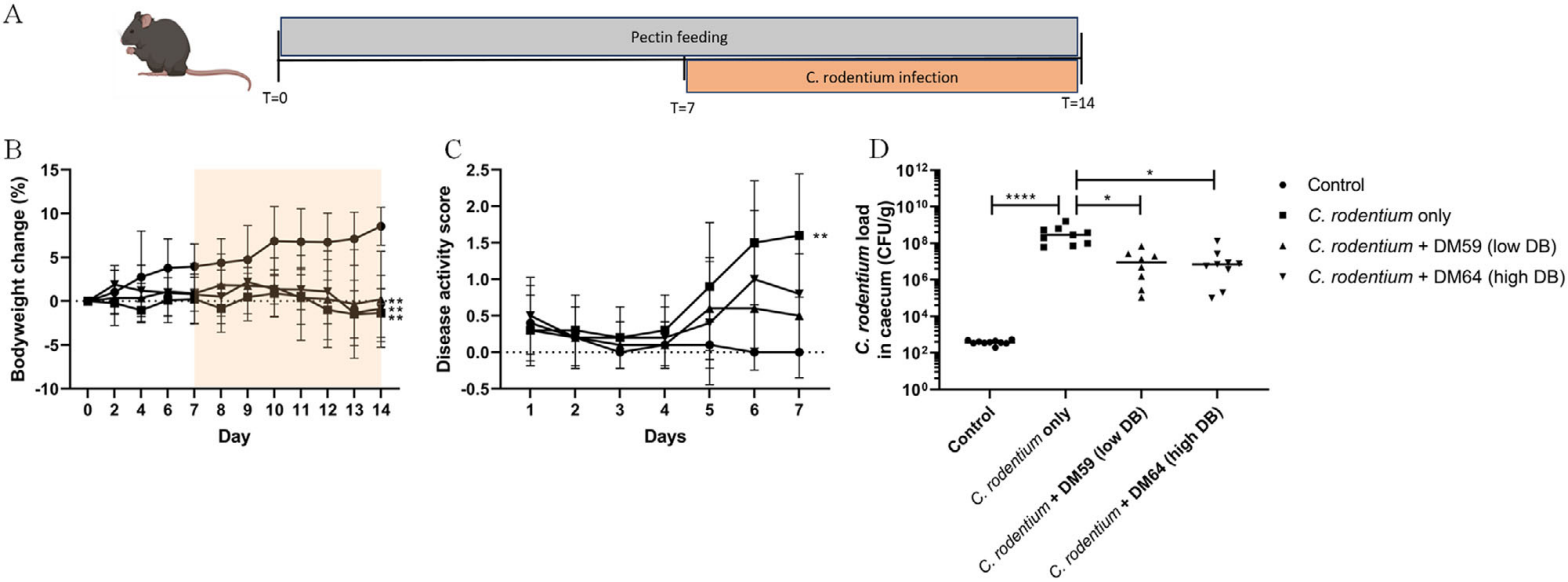
Body weight change, disease activity score and cecal *C. rodentium* load. Study design of pectin feeding and *C. rodentium* infection (A). Bodyweight (B) was monitored every two days until day 7. From day 7, after *C. rodentium* was administered, bodyweight and disease activity score (C) were monitored daily. At day 14, mice were sacrificed and *C. rodentium* load in caecal digesta was determined with PCR (D). Statistical differences between control and other experimental groups from the area under the curve of the different experimental groups were determined using one‐way ANOVA, followed by Dunnet post‐test (* *p* < 0.05, ** *p* < 0.01, *** *p* < 0.001, and **** *p* < 0.0001). *n* = 10 per experimental group.

The impact of supplementation of low DB and high DB pectin structures on *C. rodentium*‐induced colitis was investigated. Mice received pectins for 14 days and at day 7 after start of pectin supplementation (**Figure** **1**A), *C. rodentium* was orally administered to mice.

Bodyweight increased in control mice during the experiment (Figure 1B), which was not observed in mice receiving *C. rodentium* nor in mice receiving *C. rodentium* + pectins (*p *< 0.01 from AUC). Disease activity score gradually increased after administration of *C. rodentium* (Figure 1C). However, mice treated with *C. rodentium* + DM59 (low DB) pectin or mice treated with *C rodentium* + DM64 (high DB) pectin showed a lower disease activity score than mice treated with *C. rodentium*. The disease activity score did not significantly differ from control mice. These results corroborated the findings on bacterial load of *C. rodentium* in caecal digesta samples (Figure 1D). *C. rodentium* treated mice had a high load of 4.31×10^8^ CFU/g (*p* < 0.0001) in caecal digesta, whereas *C. rodentium* + DM59 (low DB) pectin treated mice had a lower *C. rodentium* load of 1.68×10^7 ^CFU/g (*p* < 0.05 vs *C. rodentium* treatment) while *C. rodentium* + DM64 (high DB) pectin treated mice had a lower *C. rodentium* load of 2.14×10^7 ^CFU/g (*p* < 0.05 vs *C. rodentium* treatment). Thus, low and high DB pectin treatments both resulted in a lower disease activity score and lower *C. rodentium* load.

**Figure 2:**
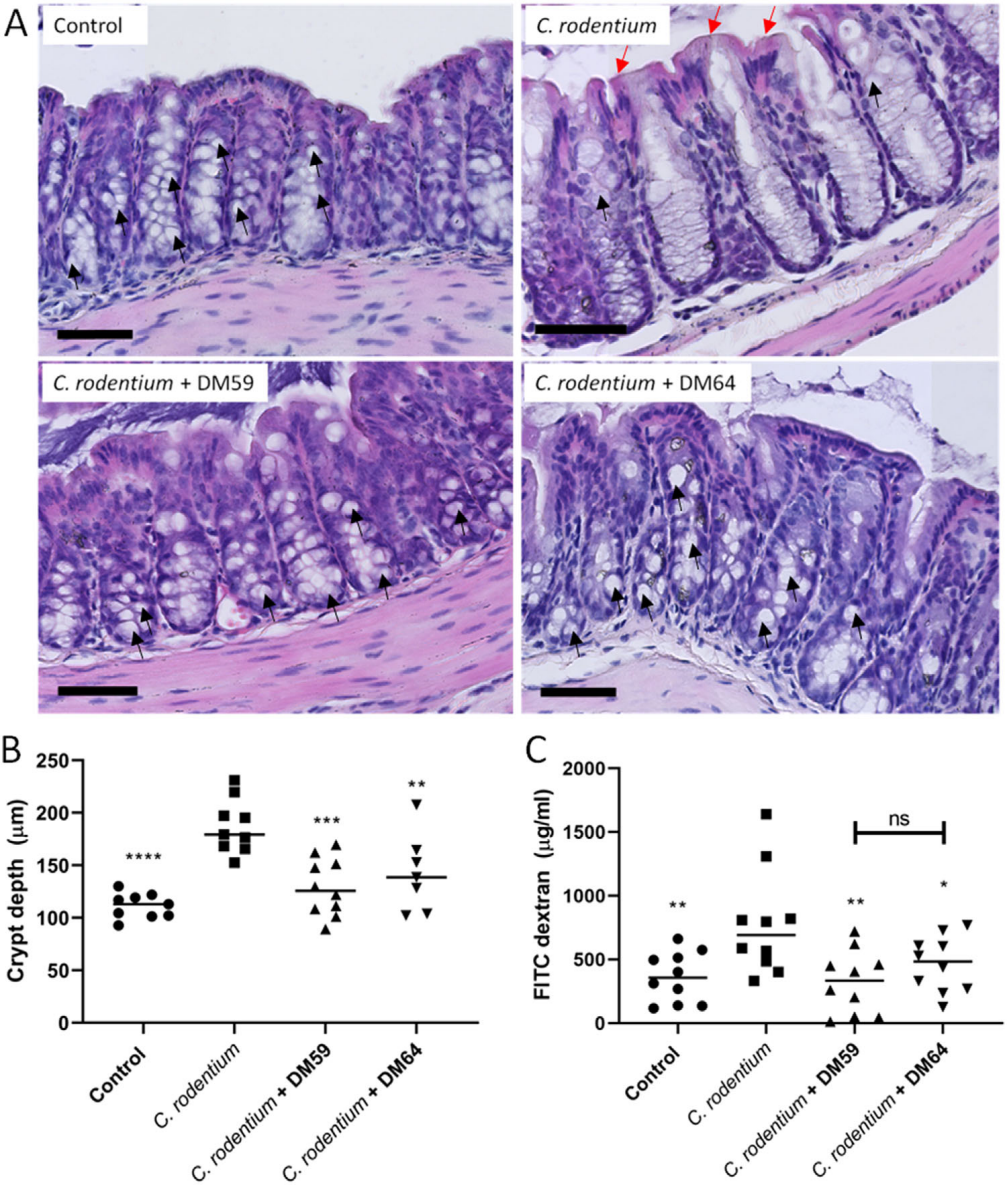
The protective impact of pectins on *C. rodentium*‐induced barrier disruption. Colon sections from mice were stained with H&E staining (A). From these sections, crypt depth was quantified to determine crypt hyperplasia (B). From plasma, FITC dextran flux was measured to determine intestinal permeability (C). Statistical differences between control and other experimental groups were determined using one‐way ANOVA, followed by Dunnet post‐test (* *p* < 0.05, ** *p* < 0.01, *** *p* < 0.001, and **** *p* < 0.0001). Black arrows indicate goblet cells and red arrows indicate epithelial damage. Scale bar = 70 µm.

Next, the protective effect of pectins was investigated on *C. rodentium*‐induced colonic damage and *C. rodentium*‐induced intestinal barrier dysfunction. Histological analysis (**Figure** **2**A) of the colon revealed that *C. rodentium* induced severe intestinal damage that is characterized by goblet cell depletion, epithelial damage, and increased crypt length (Figure 2B). *C. rodentium* induced a crypt length of 187.3 µm which was higher (*p < *0.0001) than in control mice which had a crypt length of 111.4 µm. Pectins protected from this *C. rodentium*‐induced crypt elongation as *C. rodentium* + DM59 (low DB) treated mice had a crypt length of 129.3 µm (*p < *0.01 compared to *C. rodentium*) and *C. rodentium* + DM64 (high DB) pectin treated mice showed a crypt length of 142.7 µm (*p < *0.05 compared to *C. rodentium*). These protective effects were not different between the pectins.

Intestinal barrier function disruption was also beneficially influenced by the pectins (Figure 2C). *C. rodentium* treated mice had a plasma FITC‐dextran concentration of 776.6 µg/ml which was significantly (*p* < 0.01) higher than the 362.6 µg/ml of control mice. The *C. rodentium* + DM59 (low DB) pectin treated mice and *C. rodentium* + DM64 (high DB) pectin treated mice had a significant lower concentration of plasma FITC‐dextran of respectively 323.4 µg/ml (*p* < 0.01 vs *C. rodentium*) and 464.8 µg/ml (*p* < 0.05 vs *C. rodentium*). These results suggest together that both low and high DB pectins prevent *C. rodentium*‐induced colonic damage and attenuate *C. rodentium*‐induced intestinal barrier dysfunction.

### 3.3 Pectins Prevent a *C. rodentium*‐Induced Increase of pro‐Inflammatory T cell Subsets, but they do not Attenuate *C. rodentium*‐Induced Cytokine Secretion in the Caecum

**Figure 3:**
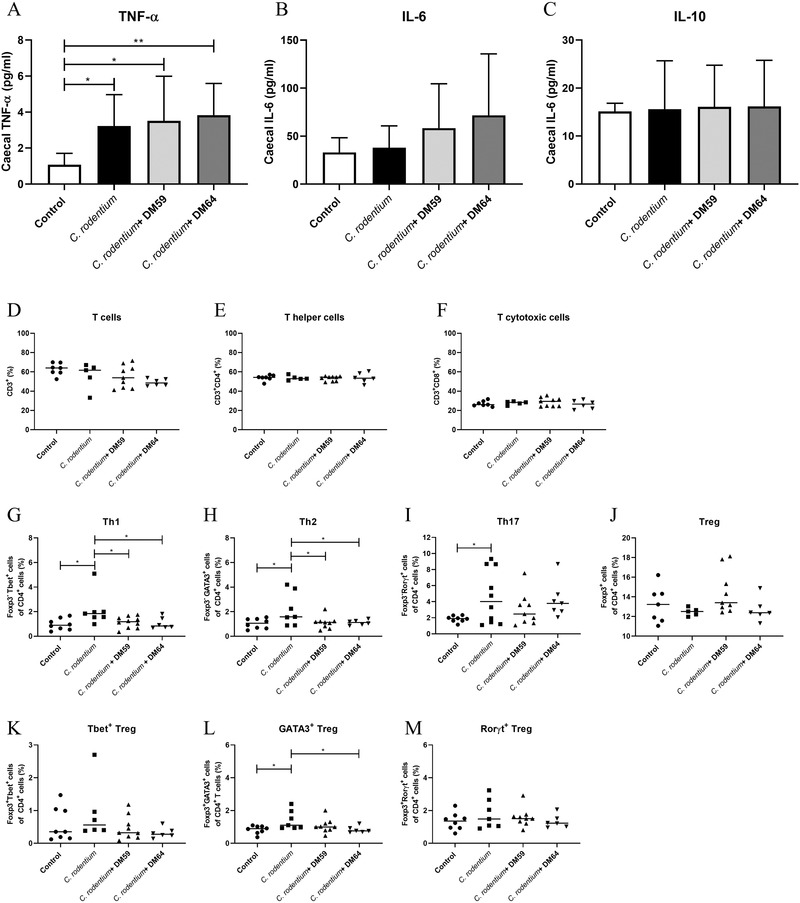
The protective impact of DM59 (low DB) and DM64 (high B) pectins on *C. rodentium*‐induced immune responses. TNF‐α (A), IL‐6 (B), and IL‐10 levels (C) were determined from caecal lysates from mice. Percentages of T cells (D), helper T cells (E), cytotoxic T cells (F), Th1 cells (G), Th2 cells (H), Th17 (I), regulatory T cells (J), Tbet^+^ Tregs (K), GATA3^+^ Tregs (L), and Rorγt^+^ Tregs (M) in the MLN of control or pectin supplemented mice. Statistical differences between experimental groups were determined with one‐way ANOVA, followed by Dunnet post‐test (* *p* < 0.05, and ** *p* < 0.01).

Since pectins prevent *C. rodentium*‐induced intestinal damage, we investigated the impact of the different pectins on *C. rodentium*‐induced immune responses. First, levels of the cytokines IL‐6, TNF‐α, and IL‐10 from cecal tissues were determined. As shown in **Figure** **3**, *C. rodentium* induced a significant increase of 66.5% (*p* < 0.05) of cecal TNF‐α, whereas cecal IL‐6 and IL‐10 were unaffected by the *C. rodentium* infection. Pectin supplementation did not prevent this increase of TNF‐α as the DM59 (low DB) pectin‐treated mice also had a 69.2% (*p* < 0.05 compared to control) higher level and the DM64 (high DB) pectin treated mice had a 71.8% (*p* < 0.01 compared to control) higher level of cecal TNF‐α levels after *C. rodentium* infection.

The impact of pectins on *C. rodentium* induced T cell frequencies in the MLN was also investigated. T helper cell subsets were impacted by *C. rodentium* infection. *C. rodentium* increased Th1 levels with 54.9% (*p* < 0.05, Figure 3G), Th2 levels with 50.2% (*p* < 0.05, Figure 3H), and Th17 levels with 59.8% (*p* < 0.05, Figure 3I). Regulatory T cell levels were unaffected by *C. rodentium* treatment (Figure 3J). Both pectins showed a protective effect on these *C. rodentium* induced T cell frequencies. The DM59 (low DB) and DM64 (high DB) pectins treatments reduced *C. rodentium*‐induced Th1 levels with 47.2% (*p* < 0.05) and 50.4% (*p* < 0.05), respectively. A similar protective effect of both pectins was found on Th2 cells as they reduced *C. rodentium*‐induced increase in Th2 levels with 49.3% (*p* < 0.05) and 48.3% (*p *< 0.05), respectively. However, DM59 (low DB) pectin and DM64 (high DB) pectin treatment did not prevent the increase of Th17 cell frequencies induced by *C. rodentium*. The pectin treated mice did also not show a difference in Th17 cells compared with control mice, suggesting that the pectins + *C. rodentium* treatments induce an increase in Th17 cells, but not as high as *C. rodentium* treated mice. Additionally, the levels of the regulatory T cell subsets Tbet^+^ Tregs, GATA3^+^ Tregs, and Rorγt^+^ Tregs were also measured (Figure 3K‐M). Neither *C. rodentium* nor *C. rodentium* + pectin treatments affected Tbet^+^ Tregs or Rorγt^+^ Tregs levels, but *C. rodentium* increased GATA3^+^ Tregs with 40.7% (*p* < 0.05). The *C. rodentium* + DM64 (high DB) pectin treated mice had a 40.8% lower level of GATA3^+^ Tregs compared to *C. rodentium* treated mice. The *C. rodentium* + DM59 (low DB) pectin treated mice had a 24% lower level of GATA3^+^ Tregs, which is not significantly different from control and *C. rodentium* treated mice. This suggests that *C. rodentium* + DM59 (low DB) pectin treatment reduces the *C. rodentium*‐induced increase in GATA3^+^ Tregs cells, but not to such a low level as control mice.

### 3.4 Pectins Did Not Increase the Organic Acid Levels in the Caecum

**Figure 4:**
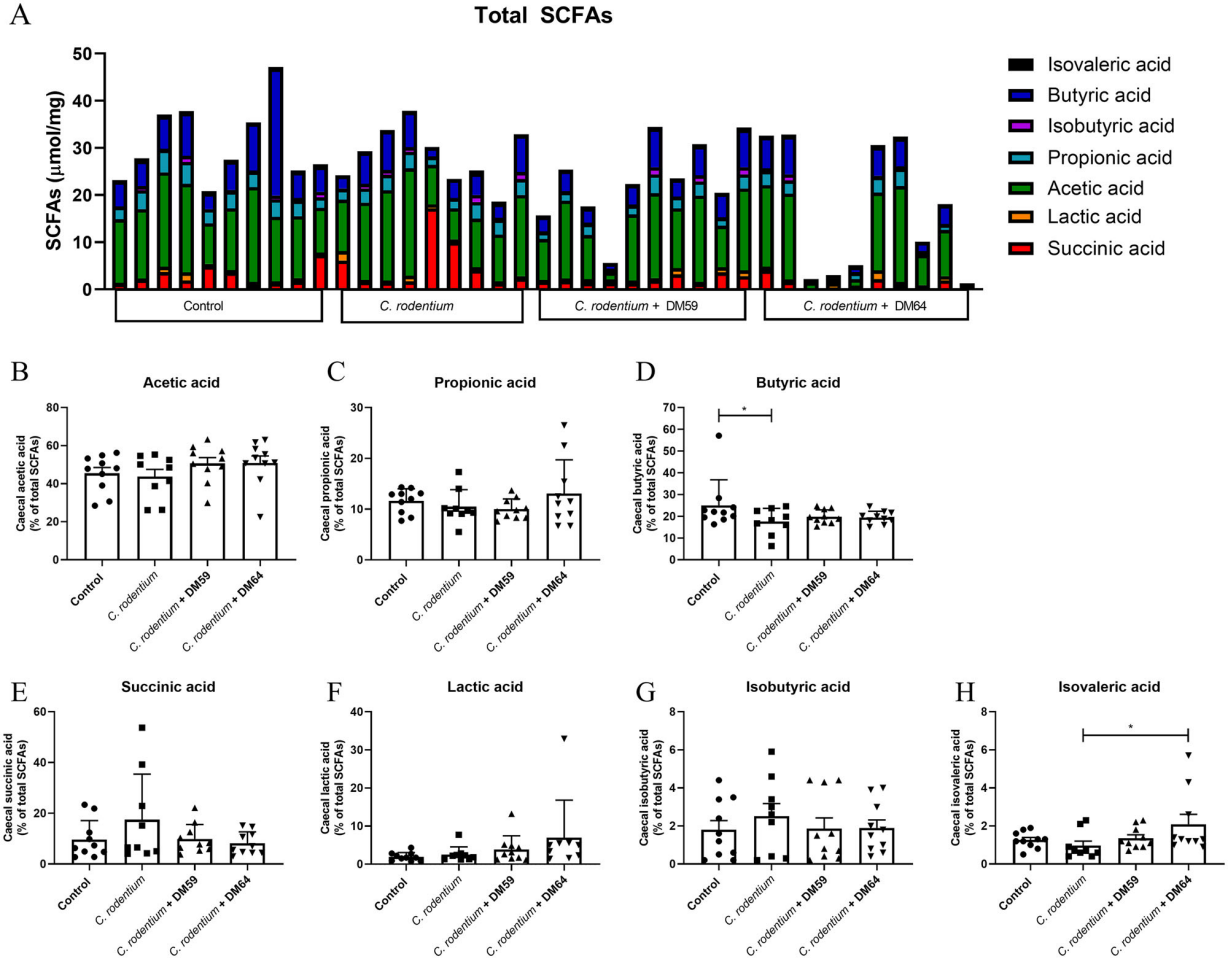
Short chain fatty acids levels in caecum of mice. Total short chain fatty acids (SCFAs) in caecum (µmol mg^−1^ dry weight of digesta) from control mice, *C. rodentium* treated mice, *C. rodentium* + DM59 (low DB) pectin treated mice or *C. rodentium* + DM64 (high DB) pectin treated mice (A). The relative abundance of acetic acid (B), propionic acid (C), butyric acid (D), succinic acid (E), lactic acid (F), isobutyric acid (G) and isovaleric acid (H) were determined from these total SCFA levels of mice. Statistical differences between experimental groups were determined using one‐way ANOVA, followed by Dunnet post‐test (* *p* < 0.05, and ** *p* < 0.01).

To investigate whether SCFAs may explain the protective effects of pectins against *C. rodentium* infection, the levels of the organic acids acetic acid, propionic acid, butyric acid, succinic acid, lactic acid, isobutyric acid, and isovaleric acid were determined in the caecum of the different mice. As shown in **Figure** **4**, both *C. rodentium* treatment and *C. rodentium* + DM59 (low DB) pectin treatment did not change total organic acid levels, whereas *C. rodentium* + DM64 (high DB) pectin treatment significantly decreased (*p* < 0.01) total organic levels in caecal digesta (Figure 4A). Most organic acids levels, including the SCFAs butyric acid and acetic acid levels were not changed by *C. rodentium* treatment or pectin + *C. rodentium* treatments. Both pectins treatments only showed a lower level of propionic acid and succinic acids compared to control and *C. rodentium* treatment, respectively (Figure 4C and 4E). These findings suggest that the protective effect against *C. rodentium* induced damage is not the result of the enhancement of SCFA or organic acid production by the intestinal microbiota.

### 3.5 Pectins Induce a Different Microbiota Composition Compared to *C. rodentium*‐Infected and Control Mice

**Figure 5:**
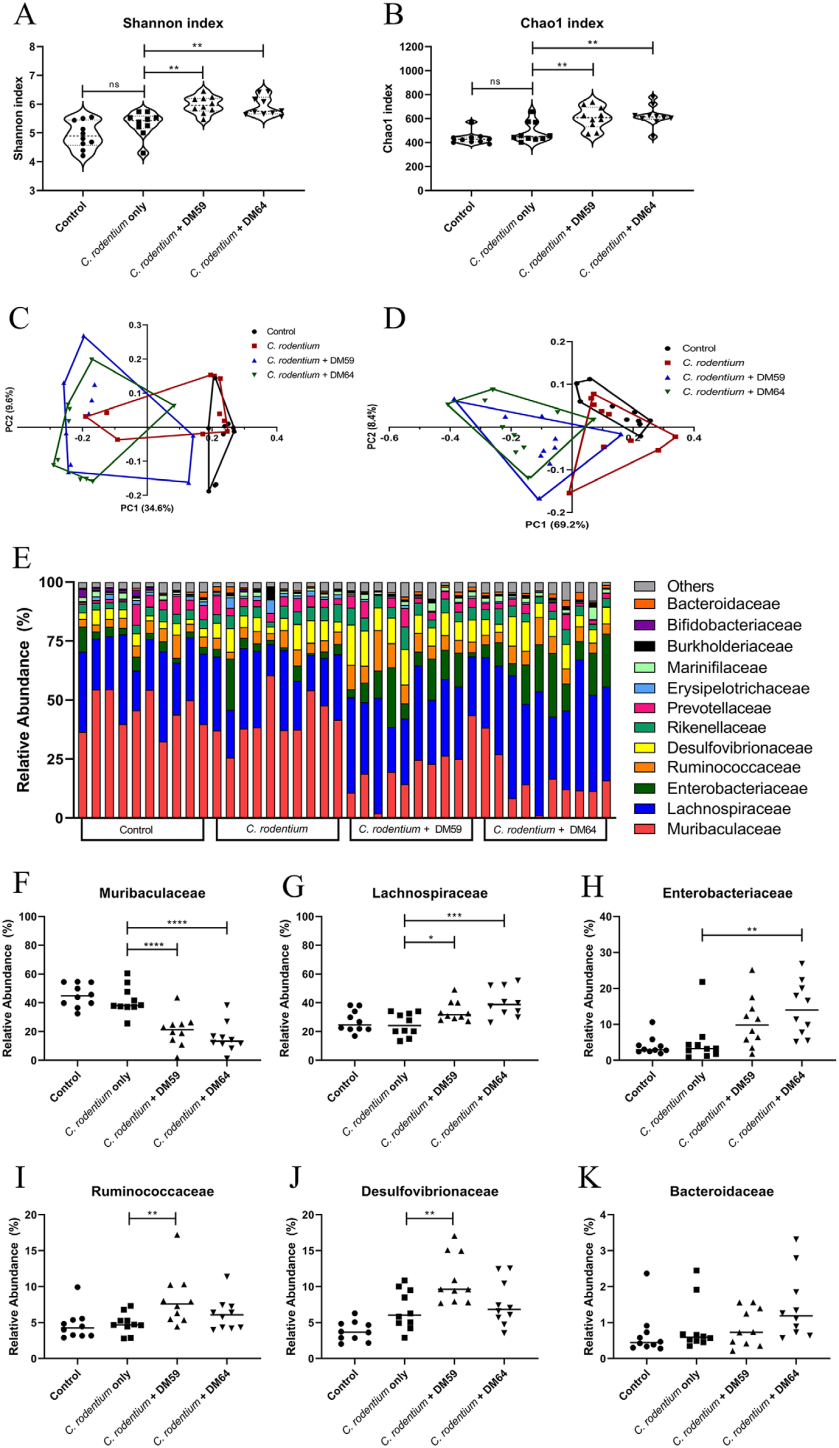
Microbiota analysis. Microbiota analysis of caecal digesta from control, *C. rodentium*, *C. rodentium* + DM59 (low DB) pectin, or *C. rodentium* + DM64 (high DB) pectin treated mice. Alpha diversity was represented with Shannon index (A) and taxa richness was represented with Chao1 index (B). PCoA plots based on unweighted (C) and weight (D) UniFrac distances of microbiome. Relative abundance of bacterial operational taxonomic units (OTUs) at family level for each individual mice (E). Presence of relative abundance of Muribaculaceae (F), Lachnospiraceae (G), Enterobacteriaceae (H), and Ruminicoccaceae (I), Desulfovibrionaceae (J), and Bacteroidaceae (K) in the caecum of the mice. Statistical differences between control and other experimental groups were determined using one‐way ANOVA, followed by Dunnet post‐test (* *p* < 0.05, ** *p* < 0.01, *** *p* < 0.001, and **** *p* < 0.0001). Significance was defined by FDR<.05.

Next, we investigated which specific changes in microbiota composition are induced by the pectins. Differences in microbiota diversity and abundance were determined with 16s RNA sequencing. As shown in **Figure** **5**, the Shannon index (Figure 5A) and Chao1 index (Figure 5B) revealed that the *C. rodentium* + DM59 (low DB) pectin treated mice and *C. rodentium* + DM64 (high DB) pectin treated mice showed higher microbiota diversity (both *p* < 0.01) and richness (both *p* < 0.01) compared to *C. rodentium*‐infected mice in absence of pectin. Additionally, PCoA plots based on unweighted (Figure 5C) and weighted (Figure 5D) UniFrac distances also revealed that treatment with both pectins resulted in a rather similar microbiota composition (Permanova, *p* > 0.05) differing both from *C. rodentium* treated mice (Permanova, *p* < 0.001) and control mice (Permanova, *p* < 0.001). These findings show that mice treated with both low or high DB pectins had a more diverse and rich microbiota composition than the *C. rodentium‐*infected mice and the controls.

Next, we investigated which bacterial groups were significantly different in abundance at the family level between the different experimental groups (Figure 5E‐I). Control mice and *C. rodentium* treated mice showed higher levels of Muribaculaceae (Figure 5F). The levels of Muribaculaceae were 21.0% (*p* < 0.0001) lower in *C. rodentium* + DM59 (low DB) pectin treated mice and 26.1% (*p* < 0.0001) in *C. rodentium* + DM64 (high DB) pectin treated mice. On the contrary, Lachnospiraceae (Figure 5G) were significantly higher in *C. rodentium* + pectin treated mice compared to *C. rodentium* treated mice. Lachnospiraceae was 9.1% (*p* < 0.05) higher in DM59 (low DB) pectin treated mice and 15.3% higher in DM64 (high DB) pectin‐treated mice (*p* < 0.001). Specific effects for *C. rodentium* + DM59 (low DB) pectin‐treated mice were also observed. *C. rodentium* + DM59 (low DB) pectin‐treated mice had a significant higher level [3.6%, (*p* < 0.01)] of Ruminococcaceae (Figure 5I) and a significant higher level [4.9%, (*p* < 0.01)] of Desulfovibrionaceae (Figure 5J) compared to mice treated with *C. rodentium* only. Typical for the *C. rodentium* + DM64 (high DB) pectin treatment was a significant higher level (9.4%, (*p* < 0.01)) of Enterobacteriaceae (Figure 5) compared to *C. rodentium* only. The level of Bacteroidaceae (Figure 5K) did not differ between the different experimental groups.

**Figure 6:**
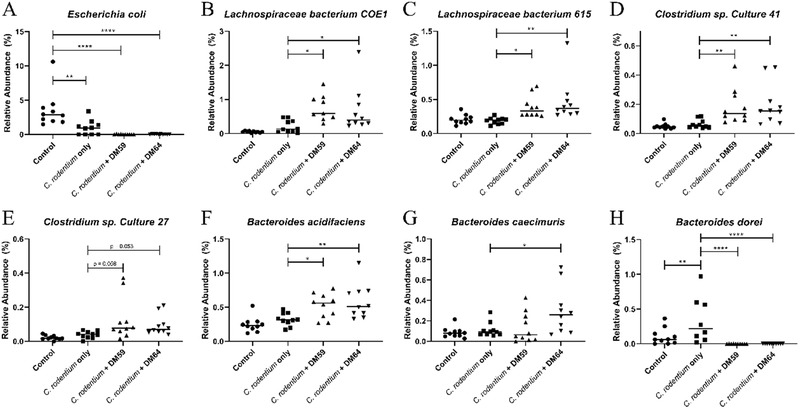
Bacterial species contributing most to variations in microbiota composition after SIMPER test. Relative abundance of *Escherichia coli* (A), *Lachnospiraceae bacterium COE1* (B), *Lachnospiraceae bacterium 615* (C), *Clostridium sp. Culture 41* (D), and *Clostridium sp. Culture 27* (E), *Bacteroides acidifaciens* (F), *Bacteroides caecimuris* (G), and *Bacteroides dorei* (H) from caecal digesta from control, *C. rodentium*, *C. rodentium* + DM59 (low DB) pectin, or *C. rodentium* + DM64 (high DB) pectin treated mice. Statistical differences between experimental groups were determined using one‐way ANOVA for parametrically distributed data and Kruskal‐Wallis for non‐parametrically distributed data, followed by Dunnet post‐test (* *p* < 0.05, ** *p* < 0.01, *** *p* < 0.001, and **** *p* < 0.0001). Significance was defined by FDR<.05.

To identify which bacterial species explain the variation in microbiota composition between *C. rodentium* and *C. rodentium* + pectin treated mice, we performed a SIMPER test (Supplementary Tables 1–S3, Supporting Information). The species contributing most to the variations in microbiota composition and showing significant differences between the experimental groups were plotted in **Figure** **6**. An increase of specific species from Lachnospiraceae and Bacteroidaceae and a decrease in species from Enterobacteriaceae and Bacteroidaceae contributed to the differences in microbiota composition between mice treated with *C. rodentium* and the pectins and mice treated with *C. rodentium* only. *E. coli*, a species from Enterobacteriaceae, was decreased in all *C. rodentium*‐treated groups compared to the control. However, various species from Lachnospiraceae, such as *Lachnospiraceae bacterium COE1* (Figure 6B), *Lachnospiraceae bacterium 615* (Figure 6C), and *Clostridium sp. Culture 41* (Figure 6D) were significantly increased in *C. rodentium* + DM59 (low DB) pectin treated mice (*p* <0.05; *p* < 0.05, *p* < 0.01, respectively) and in *C. rodentium* + DM64 (high DB) pectin‐treated mice (*p* <0.05; *p* < 0.01, *p* < 0.01, respectively) compared to *C. rodentium* treated mice. There was also a trend towards an increase in *Clostridium sp. Culture 27* (Figure 6E) in these mice. Additionally, compared to *C. rodentium* treated mice, species from Bacteroidaceae were increased, such as *Bacteroides acidifaciens* (Figure 6F) in *C. rodentium* + DM59 (low DB) pectin and *C. rodentium* + DM64 (high DB) pectin‐treated mice (respectively *p* <0.05; *p* < 0.01) and *Bacteroides caecimuris* (Figure 6E) in *C. rodentium* + DM64 (high DB) pectin‐treated mice (*p* <0.05). Another species from Bacteroidaceae, *Bacteroides dorei* (Figure 6H), was only increased in *C. rodentium* treated mice and was significant lower in control (*p* < 0.01) and *C. rodentium* + DM59 (low DB) pectin (*p* < 0.0001) and *C. rodentium* + DM64 (high DB) pectin‐treated mice (*p* < 0.0001).

## 4 Discussion

Dietary fibers are known to impact intestinal immunity through microbiota dependent ways.^[^ ^34^ ^]^ Pectin is one of these dietary fibers that is known to exert these beneficial effects,^[^ ^7^ ^]^ but it is unknown how pectins that structurally differ in the DB influence the microbiota‐dependent impact on intestinal immunity. Therefore, in the current study we explored how high DM pectins that differ in the DB exert anti‐inflammatory properties through modulating intestinal microbiota communities in a model of *C. rodentium*‐induced colitis. The current study showed that treatment with both tested pectins prevented the development of *C. rodentium*‐induced colitis in mice. This protective effect was independent of the DB of pectins. Treatment with both pectins reduced intestinal damage, preserved intestinal barrier function, and was associated with a reduced level of specific T cell subsets. These anti‐inflammatory effects of pectins were not derived from the production of SCFAs in the cecum, but may rather be explained by alterations in microbiota composition which reduced the *C. rodentium* load and there with pathogenicity in the caecum of pectin treated mice.

The lower *C. rodentium* load in low and high DB pectins treated mice may be responsible for the anti‐inflammatory effect of the pectins on the development of *C. rodentium*‐induced colitis. A lower *C. rodentium* exposure to epithelial cells avoids the attachment of *C. rodentium* to epithelial cells which protects from *C. rodentium‐*induced epithelial barrier disruption and transmissible murine colonic hyperplasia.^[^ ^11^, ^22^, ^35^ ^]^ In our study we found a reduced crypt length and a reduced barrier disruption in mice treated with both pectins, suggesting that pectins prevent the binding of *C. rodentium* to epithelial cells and prevent the development of *C. rodentium*‐induced epithelial damage. Moreover, the lower attachment of *C. rodentium* to epithelial cells may also be responsible for the limited development of colitis in these mice. Both low and high DB pectins induced anti‐inflammatory effects on *C. rodentium*‐induced colitis that was characterized by reduced levels of Th1 which are increased in *C. rodentium*‐induced colitis,^[^ ^22^ ^]^ and GATA3+ Tregs which are related to tissue inflammation.^[^ ^36^, ^37^ ^]^ There was, however, still some level of inflammation in these mice that was characterized by increased levels of TNF‐α and Th17 cells. Together, these findings suggest that pectins lower the *C. rodentium* load in the digesta which prevents *C. rodentium* attachment to epithelial cells and the induction of colonic inflammation.

The low and high DB pectins may lower the *C. rodentium* load in the digesta through several mechanisms. Pectin molecules are known to directly bind with *C. rodentium* and exert anti‐microbial effects.^[^ ^11^ ^]^ The large availability of pectins to interact with *C. rodentium* through pectin ingestion may inhibit the growth of the pathogen in the caecum. The digestion of the pectins by the intestinal microbiota may, however, change this inhibitory impact of pectin molecules on *C. rodentium* growth.^[^ ^11^ ^]^ Nevertheless, the digested pectin in the form of galacturonic acid (GalA) monomers may be able to inhibit the growth of *C. rodentium*, because GalA are known to influence the virulence of *C. rodentium* in the caecum and thereby reduce the overgrowth of the pathogen.^[^ ^38^ ^]^ Jimenez et al. ^[^ ^38^ ^]^ demonstrated that the digestion of pectins leads to the release of GalA, which are sensed by the transcription factor ExuR in *C. rodentium*.^[^ ^38^ ^]^ In absence of GalA, the ExuR transcription factor will stimulate the expression of the virulence genes of *C. rodentium* which leads to epithelial attachment and colitis. In presence of GalA, however, GalA binds to ExuR and suppresses the expression of specific virulence genes of *C. rodentium*.^[^ ^38^ ^]^ The loss of virulence factors prevents binding of *C. rodentium* to epithelium and localizes *C. rodentium* in the luminal digesta. Here, the growth of the pathogen can be outcompeted by other commensal bacteria, which leads to a lower *C. rodentium* load.^[^ ^39^, ^40^ ^]^ Overall, the pectin treatment may induce anti‐microbial effects on *C. rodentium* or prevent the expression of virulence factors by *C. rodentium*. Consequently, the growth of *C. rodentium* is outcompeted in the lumen by other commensal bacteria which reduces the *C. rodentium* load and prevents the development of *C. rodentium*‐induced colitis.

Our data showed that treatment with the low or high DB pectins induces alterations in microbiota compositions which may prevent *C. rodentium* pathogenicity and associated colitis. Pectin is known to stimulate Lachnospiraceae communities^[^ ^20^, ^21^ ^]^ as species from Lachnospiraceae possess pectin‐degrading enzymes.^[^ ^41^ ^]^ The increased abundance of Lachnospiraceae by the pectins may enhance the digestion of pectins. This leads to an enhanced galacturonic acid availability for *C. rodentium* which reduces the virulence of the pathogen and attenuates colitis.

Intestinal SCFAs are important in the regulation of immune function and intestinal homeostasis and have strong anti‐inflammatory properties on *C. rodentium*‐induce colitis.^[^ ^42^ ^]^ It is however challenging to measure SCFA accurately in the intestinal tissues due to the rapid metabolization by intestinal microbiota or intestinal tissues.^[^ ^43^ ^]^ Our data demonstrated that despite both pectins induce an increase in SCFA producing bacteria, *C. rodentium* infected mice treated with DM64 (high DB) showed lower caecal SCFA levels compared to control mice, whereas mice treated with DM59 (low DB) did not experience a lowering of SCFA. This difference in SCFA levels might be explained by a difference in fermentability between the two pectins. A previous study found that readily fermented resistant starches reduced SCFA levels at the peak infection period in *C. rodentium* infected rats, whereas slowly fermented wheat bran increased SCFA levels.^[^ ^42^ ^]^ Pectin degrading enzymes prefer pectin substrates containing large blocks of non‐esterified GalA residues to pectin substrates containing high levels of methyl esters.^[^ ^19^ ^]^ DM64 (high DB) pectin may therefore be more readily fermentable than the DM59 (low DB) pectin and may contribute to lower cecal SCFA levels in the DM64 (high DB) treated mice.

*C. rodentium* infections induce strong immune responses in the colonic tissues that are characterized by increased T cell frequencies and enhanced cytokine levels.^[^ ^22^ ^]^ Our data corroborated this finding as we observed enhanced Th17 levels in mice treated with *C. rodentium* only. Wang et al. demonstrated that this induction of Th17 during *C. rodentium* infection is strongly supported by Treg cells, because deletion of Treg cells in mice prevented the induction of Th17 cells during *C. rodentium* infection.^[^ ^44^ ^]^ However, our data did not show an enhanced level of Treg cells in *C. rodentium* infected mice, suggesting that the presence rather than an increase of Treg cells are essential for the induction of Th17 cells. Moreover, the GATA3^+^ cell frequencies were also increased in mice treated with *C. rodentium* only, but decreased in mice treated with DM64. GATA3^+^ Treg cells express ST2 by which they can sense epithelial derived IL‐33, an alarmin which is produced by intestinal epithelial cells upon infection. In response to IL‐33, ST2^+^ GATA3^+^ Treg cells get activated and expand.^[^ ^45^ ^]^ In the current study the pectins might limit *C. rodentium*‐induced epithelial damage and prevented thereby the expansion of GATA3^+^ Treg cells in mice. Furthermore, our data also showed enhanced levels of Th1 and TNF‐α in *C. rodentium* infected mice, but only Th1 and not TNF‐α levels were reduced by both pectin treatments. Previously it was demonstrated that TNF‐α was not only derived from Th1 cells,^[^ ^46^ ^]^ but also from innate immune cells, such as macrophages or dendritic cells during *C. rodentium* infection in mice.^[^ ^47^, ^48^ ^]^ These findings suggest therefore that pectins may only limit the induction of T cell responses during *C. rodentium* infection, but pectins do not prevent the induction of innate immune responses by *C. rodentium*.

In the large intestine, a thick mucus layer and the high abundance of pectin‐degrading enzymes prevent direct interactions of pectin molecules with the intestinal immune system. Here, pectins can influence intestinal immunity only through microbiota‐dependent effects. This is different for the small intestine which contains a loose mucus layer and a low abundance of microbiota that allow pectins to interact directly with the immune system.^[^ ^7^ ^]^ Which pectins impact immunity through direct effects or microbiota‐dependent effects is dependent on specific structural characteristics of pectin.^[^ ^7^ ^]^ Our data showed that the DB of pectins is not a structural characteristic that changes the microbiota‐dependent effect of high DM pectins. This in contrast to the DB‐dependent impact of ∼DM46 pectins on small‐intestinal inflammation.^[^ ^14^, ^18^ ^]^ A DM43 pectins with a high DB induces similar strong inhibiting properties on TLR2‐1 as low DM pectins, whereas a DM49 pectin with a low DB did not inhibit TLR2‐1 strongly.^[^ ^18^ ^]^ Small intestinal inflammation was prevented through direct effects of these pectin structures on TLR2‐1 and not through microbiota dependent effects.^[^ ^14^ ^]^ The microbiota‐dependent effects of pectins may rather be influenced by the DM of pectins. In the presence of microbes that contain pectin degrading enzymes, low DM pectins are easily digested by microbial enzymes due to the lack of bound methyl‐esters.^[^ ^19^ ^]^ This results in a different alteration of intestinal microbiota composition between low and high DM pectins in rats and piglets.^[^ ^20^, ^21^ ^]^

Collectively, our study demonstrates that high DM pectins with different DB equally induce anti‐inflammatory properties in mice with *C. rodentium* infection through microbiota dependent ways. Both tested pectins enhanced the diversity of the intestinal microbiota which may reduce the *C. rodentium* load in the caecum. Consequently, both pectins prevent the *C. rodentium*‐induced epithelial damage, intestinal inflammation, and intestinal damage. The pectins may induce antimicrobial effects^[^ ^11^ ^]^ on *C. rodentium* or reduce the virulence of *C. rodentium* by the interaction of GalA monomers with the transcription factor EspE.^[^ ^38^ ^]^ This knowledge on how pectin structures impact immunity through stimulation of the intestinal microbiota can be instrumental in the design of functional food applications. Consumers may profit from high DM pectin consumption as it may stimulate the diversity of the microbiota composition, which can reduce the risk of infection of *C. rodentium*, EPEC, or EHEC.

## Conflict of Interest

The authors declare no conflict of interest.

## Author Contributions

M.B., and P.d.V. designed the study. M.B., R.A, and T.K. performed the animal experiments and histology experiments. T cell staining and flow cytometry was performed by A.L assisted by R.A. Both M.B and C.K. isolated DNA for microbiota analysis. Microbiota analysis was performed by M.B. and assisted by M.M.F. E.J. and H.A.S. characterized the pectins and performed SCFA measurements. M.B., and P.d.V. wrote the article. All authors have revised and improved the manuscript.

## Supporting information

Supplementary information

Supplementary information

Supplementary information

Supplementary information

Supplementary information

## Data Availability

Research data are not shared.
