# Supplementary figures and images for: Pectins that Structurally Differ in the Distribution of Methyl‐Esters Attenuate Citrobacter rodentium‐Induced Colitis
Source: Mol Nutr Food Res. 2021 Aug 16;65(19):2100346. doi: 10.1002/mnfr.202100346 (PMC9285458; doi:10.1002/mnfr.202100346)

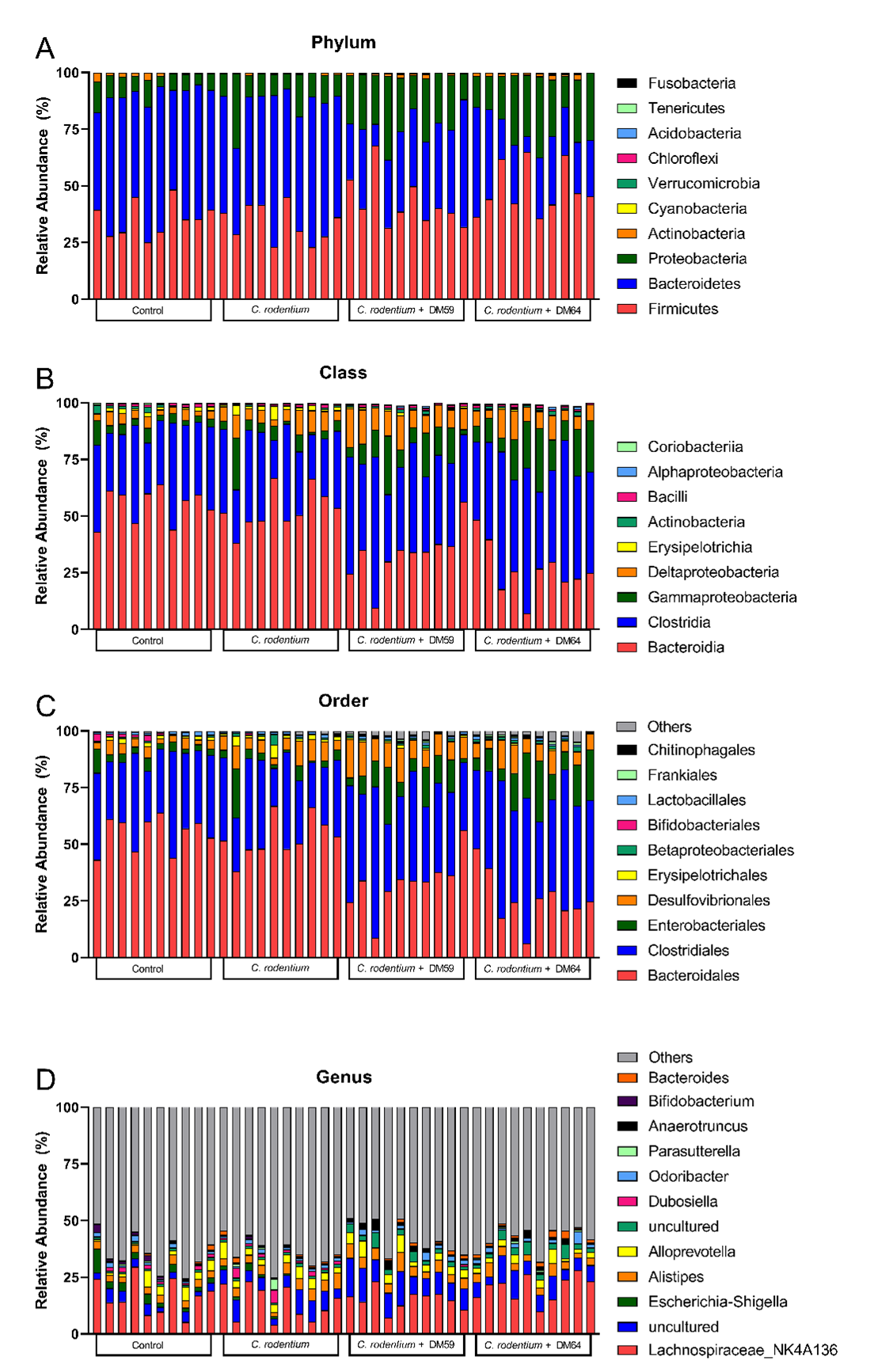

Supplement: Supplementary file 2 — Supplementary information [file MNFR-65-0-s004.tif]

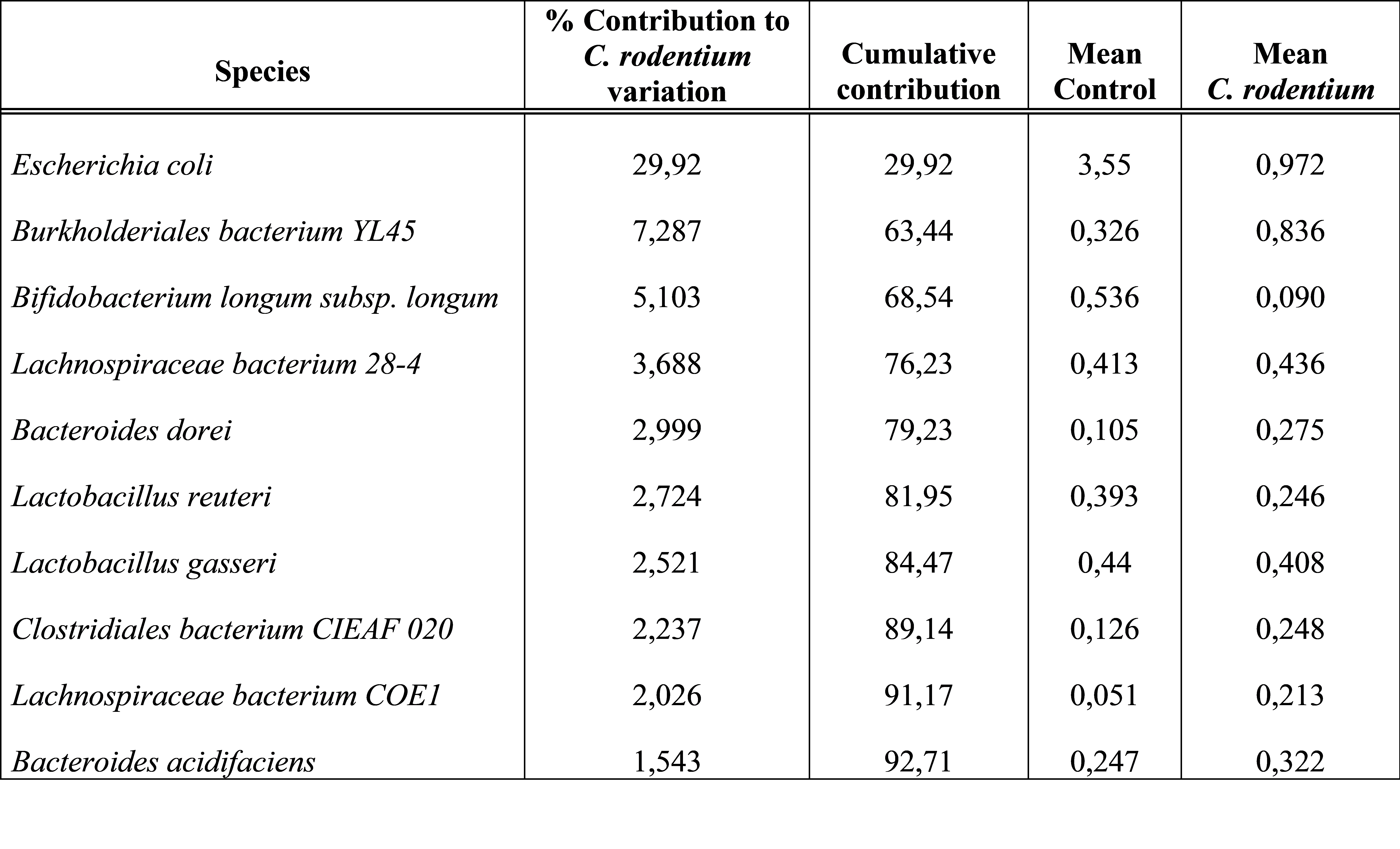

Supplement: Supplementary file 3 — Supplementary information [file MNFR-65-0-s003.tif]

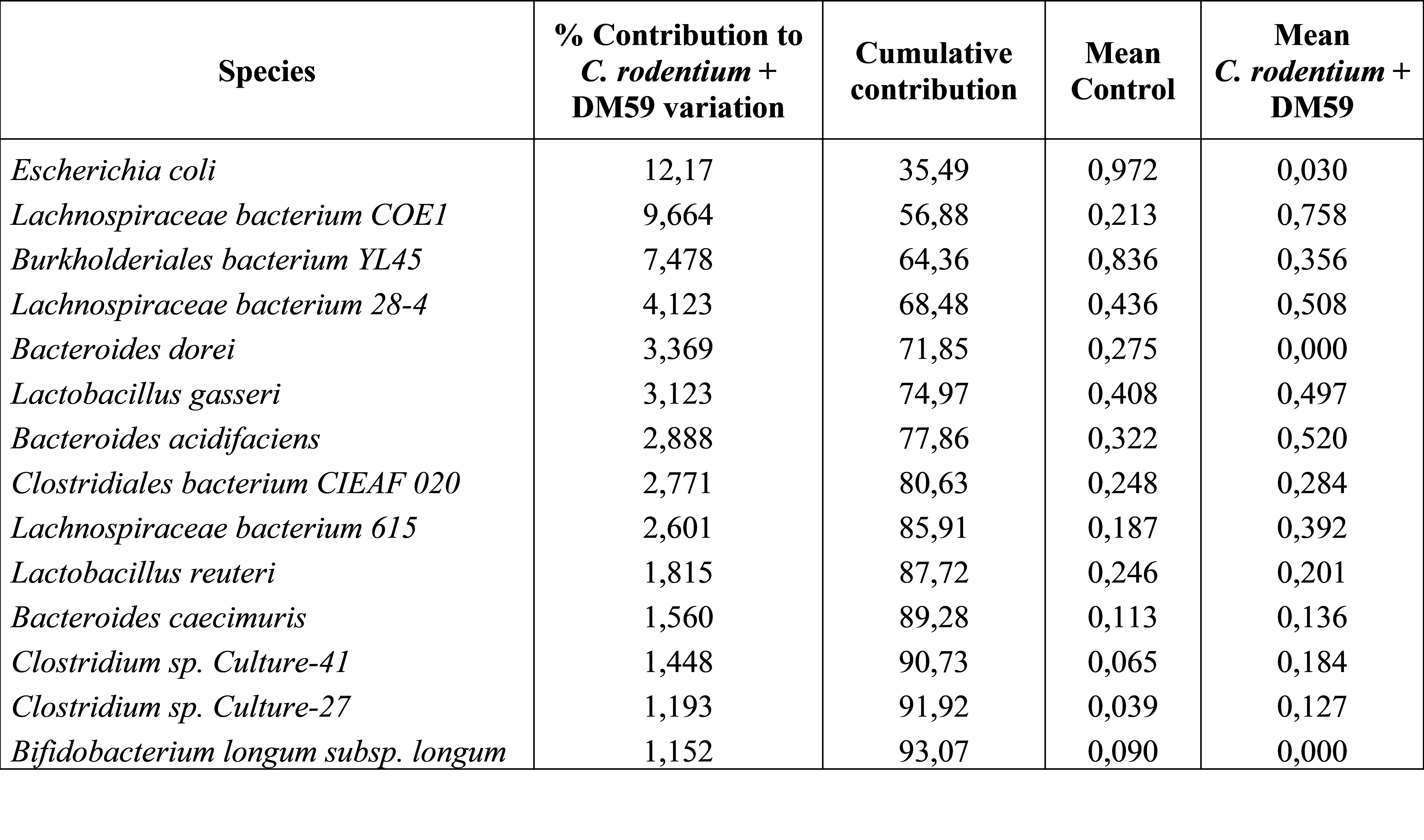

Supplement: Supplementary file 4 — Supplementary information [file MNFR-65-0-s005.tif]

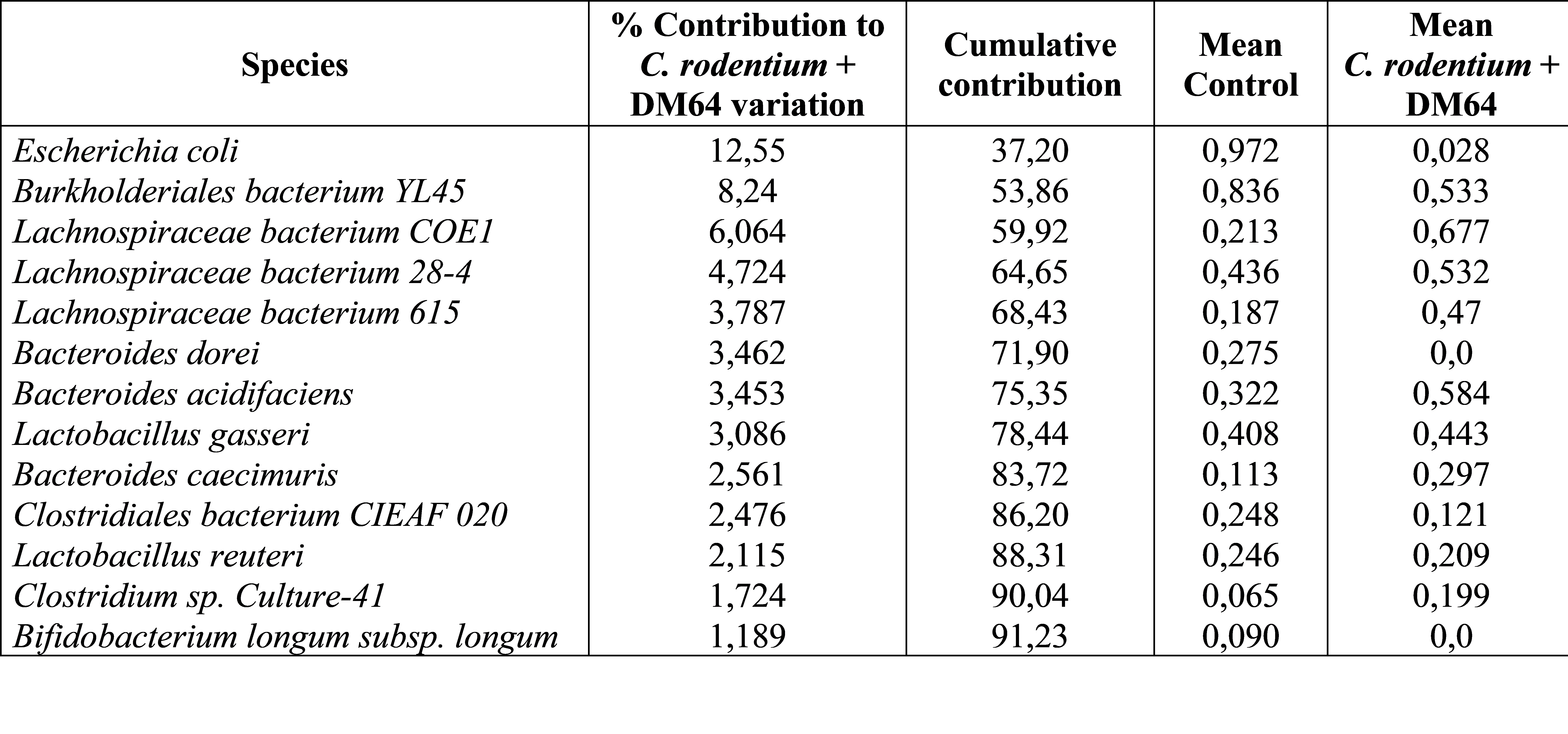

Supplement: Supplementary file 5 — Supplementary information [file MNFR-65-0-s002.tif]
